# Supplementary material for: AIBP Regulates Metabolism of Ketone and Lipids but Not Mitochondrial Respiration
Source: Cells. 2022 Nov 17;11(22):3643. doi: 10.3390/cells11223643 (PMC9688289; doi:10.3390/cells11223643)
Supplement: Supplementary file 1 [file cells-11-03643-s001.zip › cells-2006130-supplementary.pdf]

Supplementary Materials

AIBP regulates metabolism of ketone and lipids but not mitochondrial respiration

Jun-dae Kim [1,†], Teng Zhou [1,†], Aijun Zhang [2,3], Shumin Li [2], Anisha A. Gupte [2,3], Dale J. Hamilton [2,3,4] and Longhou Fang [1,3,4,*]

[1] Center for Cardiovascular Regeneration, Department of Cardiovascular Sciences, Houston Methodist Research Institute, 6550 Fannin St., Houston, TX 77030, USA
[2] Center for Bioenergetics, Houston Methodist Research Institute, 6550 Fannin St., Houston, TX 77030, USA
[3] Department of Medicine, Houston Methodist, Weill Cornell Medicine Affiliate, 6550 Fannin St., Houston, TX 77030, USA
[4] Weill Cornell Medical College, Cornell University, 407 E 61st St., New York, NY 10065, USA
* Correspondence: lhfang@houstonmethodist.org; Tel.: +713-363-9012; Fax: +713-363-9782
† These authors contributed equally to this work.

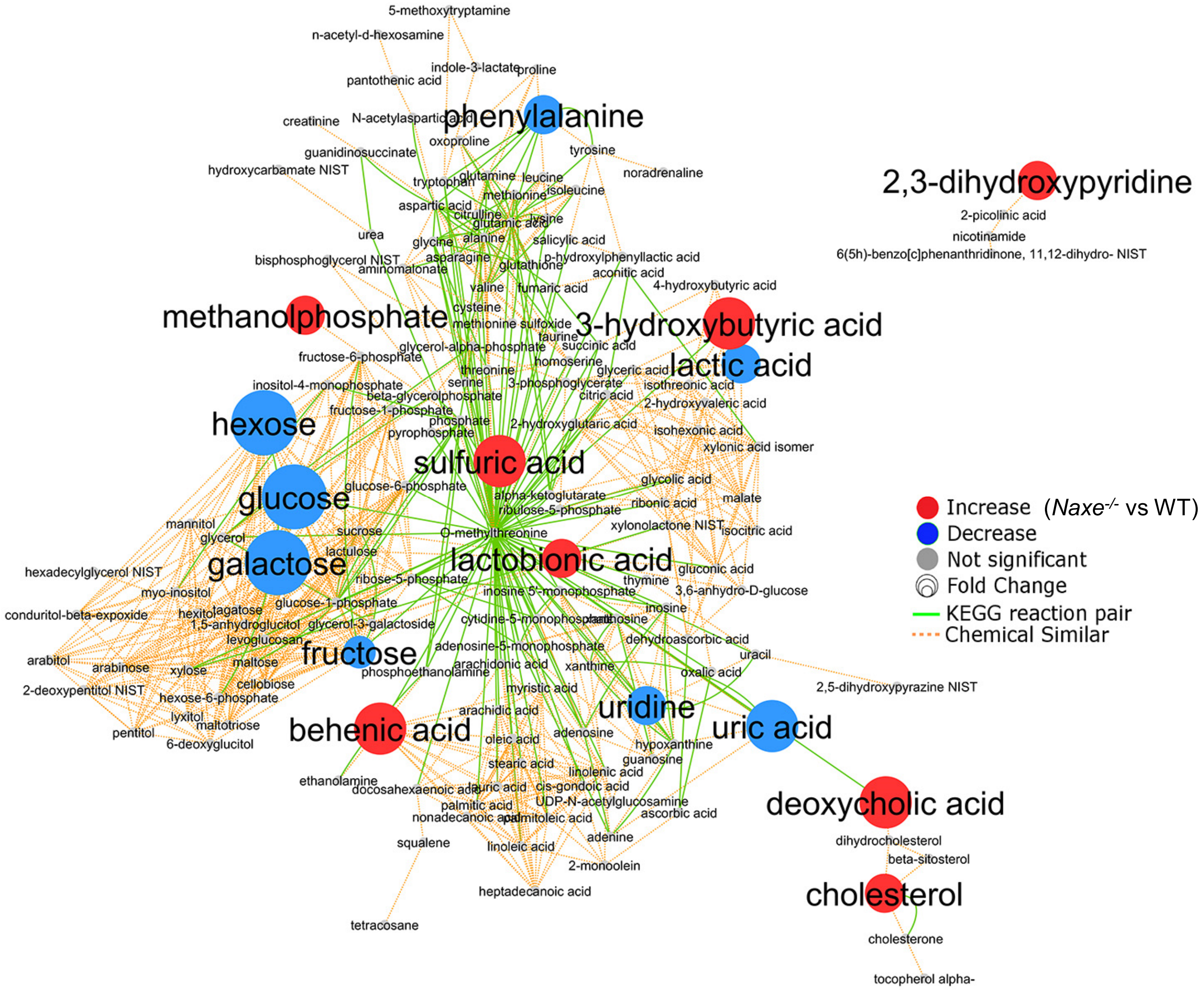

Figure S1. Bioinformatics analysis highlighting AIBP-regulated metabolites. The metabolites were analyzed at the west coast metabolomics center of UC Davis using their standard bioinformatics pipeline.

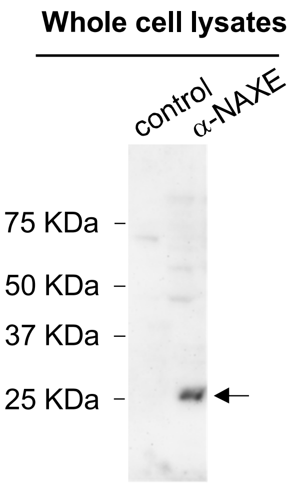

Figure S2. Western blot analysis of HUVEC cell lysates using our home-made AIBP antibody. HUVEC lysates were run on the gradient SDS PAGE, transferred to PVDF membrane, and immunoblotted using the purified anti-human NAXE antibody that was raised in rabbit.

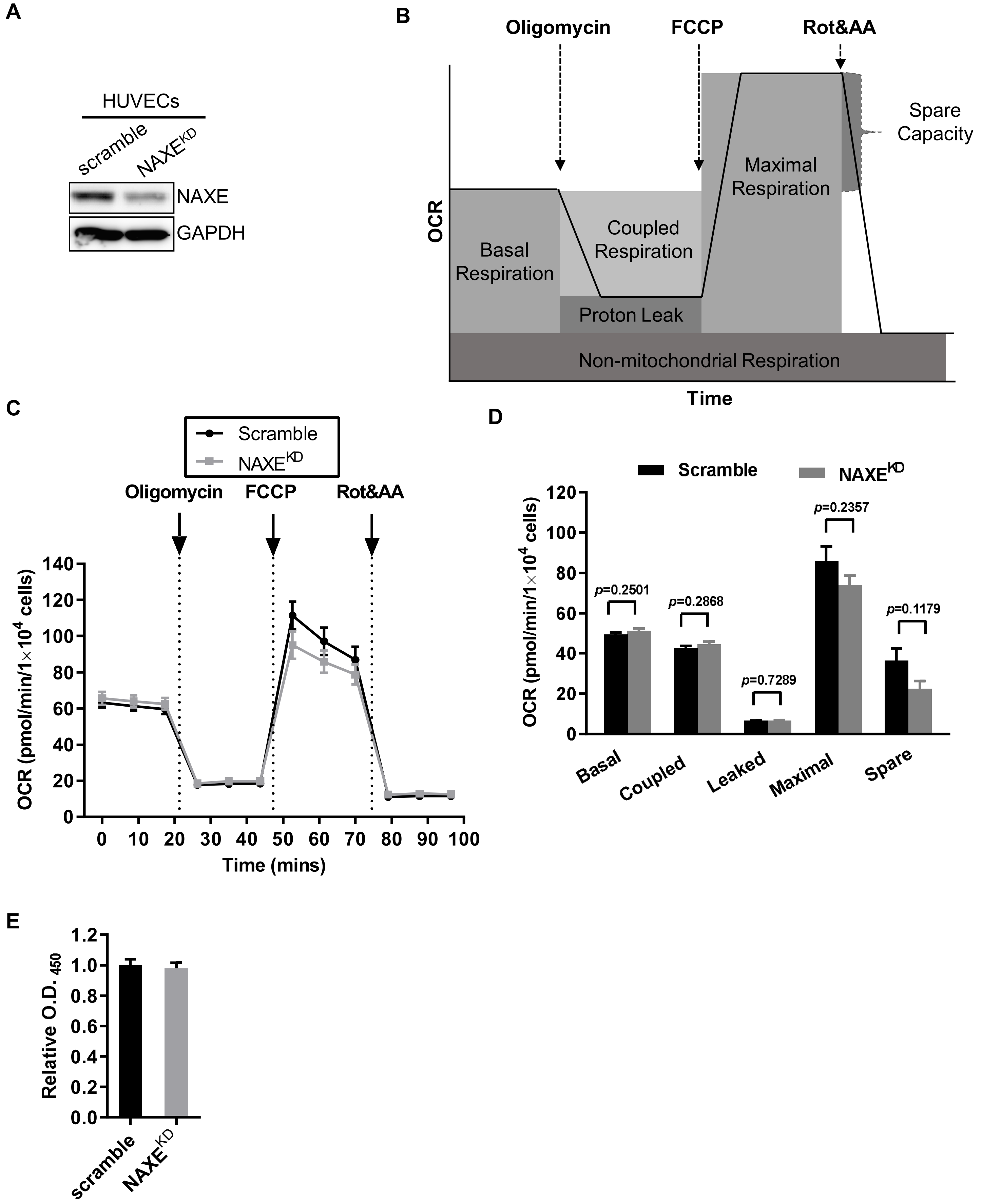

Figure S3. Mitochondrial respiration analysis of control and NAXE-deficient HUVECs using the Seahorse assay. A. Western blot analysis of NAXE expression in control or NAXE knockdown (KD) HUVECs. **B.** Diagram illustrating the seahorse assay protocol. **C.** Measurements of oxygen consumption rate (OCR) in control and NAXE KD HUVECs. **D.** Bar chart showing the mitochondrial respiration changes. **E.** Analysis of cellular dehydrogenase activity using WST-8 colorimetric assay with a CCK-8 Assay.
